# How Nurses Apply Patterns of Knowing in Clinical Practice: A Grounded Theory Study

**DOI:** 10.4314/ejhs.v31i1.16

**Published:** 2021-01

**Authors:** Forough Rafii, Alireza Nikbakht Nasrabadi, Fereshteh Javaheri Tehrani

**Affiliations:** 1 Professor, Nursing Care Research Center, Iran University of Medical Sciences, Tehran, Iran; 2 Professor, Dean, School of Nursing and Midwifery, Tehran University of Medical Sciences, Tehran, Iran; 3* Correspondence: PhD of Nursing, Nursing Care Research Center, Iran University of Medical Sciences, Tehran, Iran

**Keywords:** Nursing Care, Knowledge discovery, Patterns of Knowing, Clinical Practice

## Abstract

**Background:**

Nurses require a great deal of knowledge to provide a comprehensive and effective nursing care. A number of patterns have been put into place to help nurses acquire this knowledge. The aim of this study was to describe the core variable in the process of using patterns of knowing by nurses in clinical practice.

**Methods:**

The study was conducted in qualitative and grounded theory approach, between April 2018 and January 2020. Semi-structured interviews were used for data collection. All the interviews were transcribed verbatim. Nineteen clinical nurses were interviewed, and eight observation sessions were conducted in different hospital departments. Participants were first selected through purposeful and then theoretical sampling. Data were analyzed and interpreted using constant comparison analysis approach.

**Results:**

The findings of the study indicated that nurses apply the patterns of knowing in three ways in their clinical practice: “cohesion of patterns of knowing”, “domination of some patterns of knowing” and “elimination of some patterns of knowing”. The core variable of this process is cohesion of patterns of knowing in the domain of flexibility.

**Conclusion:**

The findings of the present study indicate that application of patterns of knowing is practiced in a range of nurse flexibility in clinical settings.

## Introduction

In the 1970s, Carper (1) introduced fundamental patterns of knowing in nursing including empirical, aesthetic, personal, and ethical knowing (2). Her work marked the beginning of a new phase of thinking among nursing researchers (3). Basic patterns of knowing encouraged nurses to identify the art of nursing work and the importance of understanding the complex nature of nursing practice (4). Empirical knowing is formally expressed through facts, models, theories, and thematic descriptions. Ethical knowing focuses on the ethical components of nursing practice and tries to answer the question of what is right and what is responsible (5). Personal knowing enables the nurse to identify his/her responses, strengths and weaknesses in a situation and to be aware of the individual biases affecting the quality of the nurse-patient relationship (6). Aesthetic knowing or the art of nursing is achieved through empathy, dynamic adaptation and understanding of the components as a whole as well as the recognition of specific cases rather than holism (1). Emancipatory knowing as a fifth patterns of knowing is a capacity for man to be aware of the society, culture and political situation of society and to critically reflect on these issues. This critical awareness and reflection is needed for change towards social justice (5).

According to Carper, each pattern of knowing is necessary but not sufficient to achieve nursing goals (1), because nursing profession involves the processes, dynamics of action, and interactions that will be effective when the five patterns of knowing come together, in which case praxis is formed. On the other hand, when one of the patterns of knowing is developed separately and regardless to the whole of knowing, it leads to uncritical acceptance, superficial interpretation, and relative use of the patterns of knowing that are called “patterns gone wild” (5). When nursing care is based solely on objective and factual knowledge, it may not be safe, and maybe of lower quality (6). Scientific knowledge of nursing has been developed over time and based on clinical experiences. Therefore, practice without theory is less likely to succeed (7).

Despite the importance of patterns of knowing in nursing, few studies have been conducted on this, in the nursing literature. The findings of one study showed that nurses often use a combination of patterns of knowing in decision-making (8). In another study, the findings indicated that patterns of knowing are interrelated and influenced by each other (9). Another study found that narrations enable nurses to discover the differences between various types of knowing and their impact on nursing practice (10). The aim of this study was to describe the core variable of the process of using patterns of knowing by nurses in clinical practice.

## Methods

**Design**: The study is part of a larger grounded theory study entitled “The process of applying patterns of knowing in the clinical practice by nurses: proposing a theoretical model” that used a qualitative research approach and grounded theory method (11–12).

**Setting**: The study was done in five different hospitals in Tehran, capital city of Iran. Two hospitals were private and the others were public. The observations and interviews were done in different hospital wards.

**Participants**: In the present study, purposive sampling and then theoretical sampling were used until saturation of data was reached (13). The researcher already knew some of the participants, and the others were selected through the observations or introduced by the other nurses. The characteristics of 19 interviewees are presented in Table 1.

**Table 1:** Demographics of the participants

**Sex**	Male: 7
	Female:12

**Education**	BSN:13
	MSN: 4
	PhD: 2
**Age**	23–54 y with average of 36/57
**Nursing** **experience**	6 months to 28 years with average of 11/97 y
**Ward**	CCU, Internal ICU, Open Heart ICU, Cardiology, Burn, Pediatric, Orology, Kidney Transplant, Emergency, , Endoscopy, Internal, General surgery
**Hospital** **type**	Public: 16
	Private: 3
**Work** **shift:**	Rotational (morning, evening, night): 14
	Fixed (morning and evening):2
	Fixed (night): 3

**Data collection**: Knowing, as a type of knowledge, is developed through nurses' experiences and their reflections; therefore, in the present study, interview and observation were selected as data collection methods with the aim of data triangulation (14). Nineteen semi-structured, deep, face-to-face and private interviews were conducted with 19 clinical nurses at the agreed time and places. All interviews were audio taped with the permission of participants.

The first question asked by the interviewer was “Describe a care situation where you used knowledge and thinking at the moment.” Then, subsequent questions were asked based on the participants' responses. In order to keep up with the participants, the researcher obtained their contact information and six short additional interviews were conducted via telephone and network communications. The interviews were returned to the participants to correct any possible errors. Duration of the interviews varied from 50 to 128 minutes.

Observations were made in the wards of Burn, Urology, Emergency, Internal Medicine, ICU of Internal Medicine and Post-CCU. In each session, important events including the way of caring, nurse-patient and nurse-companion communication, and researcher interpretations were recorded as field notes (13). Given that the grounded theory focuses on discovering social processes and individuals' interactions, the role of observer as participant was used to engage the participants. The researcher collaborated with nurses to answer patient questions, educate patients based on the educational manuals available in the ward, obtain vital signs, assist with medication, blood glucose checks, and assist with patient transfers (15).

Theoretical sampling based on the gender of the nurse, his/her education, work experience (novice or experienced), the ward in which the nurse is working and the type of hospital (private or public) occurred after initial data analysis (16). The sample size was defined through theoretical saturation.

**Data analysis**: The third author (FJT) coded and categorized each interview or observation and the first (FR) and second (ANN) authors rechecked it again. The researchers also used memoing during coding to identify the relationship between the codes and concepts (16). Having transcribed interviews and observations, data analysis has been manually analyzed using WORD software. The obtained data were constantly analyzed through constant comparison analysis method and with the researchers' theoretical sensitivity (11). Theoretical sensitivity means that the researcher can distinguish between important and trivial data and find a view toward their meanings. The researcher's professional and personal experience can be a source of information and help the sensitivity of researcher (17). The data analysis was done through four nonlinear steps: comparison of quotes or events relevant with each category, integration of categories and subcategories, theory specification and writing (11). To that end, questions starting with “What? When? Where? Why? How? What Consequence?” were asked about the data (18), (19).

**Scientific accuracy and validity of the data:** Guba and Lincoln's criteria for evaluating the quality of qualitative studies (20) were used in this study. Some experts of grounded theory studied the findings to obtain the credibility. The researcher tried to spend more time on observing and interviewing. For obtaining credibility of the results we also used member checking methods and participants' control of categories. The dependability of the findings was based on the audio and textual storage of interviews, notes, memoing, codes, categories, stories, and auditing techniques with the help of specialists. For transferability of the findings, the researcher used field notes and full description of the field under study. For conformability of the findings, the researcher attempted to be aware of her own biases and tried to limit subjectivity (14).

**Ethical consideration**: The present grounded theory study was approved by institutional research committee with code of IR.IUMS.FMD.REC.1396All. All participants signed informed consent form. All the recorded voice and the participants' information were confidential and only accessible to the research group. They were told that, they have the right to leave the study at any time without any problems.

## Results

The findings of the study indicated that nurses apply patterns of knowing in three ways in their clinical practice: “cohesion of patterns of knowing”, “domination of some patterns of knowing” and “elimination of some patterns of knowing”. The core variable of this process is “cohesion of patterns of knowing in the domain of flexibility”. All the subcategories related to the core variable are discussed below:

**Simultaneousness of knowing and flexibility**: The findings of the present study indicate that if patterns of knowing are integrated with flexibility, it will lead to appropriate patients' care and praxis.

Participant C said: *“One time we had a patient who was taking insulin. At 5 AM, I called him many times but he didn't answer. I made a painful stimulation, but he didn't answer again. I told myself that the patient probably went into hypoglycemic shock; therefore, we checked his glucose level with a glucometer. Blood glucose was forty; therefore, we called the residents, no resident answered. I saw if I wait, the patient may go into coma, so we gave him 50% dextrose. After a short time, the patient got conscious and got better.”*.

Participant D said: *“I saw many times that one of my colleagues of whom I have a very positive view about her, was educating the patient or his/her companion separately, even one or two hours after her shift time.”*

**Domination of inflexibility over context**: Sometimes nurse's inflexibility becomes more pronounced so that the nurse insists on personal values, routines, scope and timing of tasks.

Participant A said: “*We had a patient who had to undergo emergency angiography. Therefore, her groin must have been shaved. However, because her stomach was so large that she couldn't do it herself, we had no nurse assistant, I was in charge of the shift that night, and novice co-worker was with me. I told her to do this job. She said, “It's not my duty. It's the duty of nurse assistants”*. Another participant said: *“If the patient has a fever or other problems and my shift time is over, I inform it to other colleague or head nurse to notify his/her physician.”*

**Elimination of flexibility**: In some cases, elimination of flexibility is more pronounced and prominent which includes dealing with patient values, in law enforcement and in communication with patients.

Participant B said *“I have no faith in these green blessing strips that kids cover round their hands. Whenever I have to get a baby's vein, I tell his/her mom to open it. If the mom resists, I myself cut it by scissors and give it to the mom with no more word.”*

Participant G said *“At a time, a man came to visit the patient. He called the ward from the lobby. I said, “I'm sorry I can't let you visit the patient”. But he started to shout at me on the phone. I said “I won't let you visit the patient at all” and then, disconnected the conversation.”*

**Core variable**: The finding of the study indicated that nurses, sometimes such as critical situations, have the most flexibility and apply a combination of the five fundamental patterns of knowing (empirical, personal, ethical, aesthetic, and emancipatory) in their clinical practice which are appropriate to the context of care. However, sometimes, they may apply some patterns of knowing dominantly. In such situations, the nurse employs little flexibility and insists on his/her values, beliefs, routines, and timing and scope of tasks, and provides care accompanied with domination of personal knowing. According to the findings, nurses may also eliminate flexibility and one or more patterns of knowing, therefore provide ugly, unethical, and unscientific care, which is not accompanied by therapeutic communication. As it is clear, the “cohesion in patterns of knowing in domain of flexibility” is core variable in the process of applying patterns of knowing in nursing practice (Table 2).

**Table 2:** Reflective coding matrix

Central Variable: Cohesion in Patterns of Knowing in Domain of Flexibility			
**Processes**	Applying cohesion in patterns of knowing	Applying domination of some patterns of knowing	Not applying some patterns of knowing
**Characteristics**	Considering all patterns of knowing in the context of care	Prioritization of some patterns of knowing separate from care context	Elimination of some patterns of knowing separate from care context
**Dimensions**	Simultaneous application of knowledge alongside thinking, experience, context, therapeutic communication, ethics, rule, Justice and flexibility	Giving centeredness to self in different care situations of inflexibility	Elimination of scientific principles from care, elimination of therapeutic communication from care, Elimination of social justice from care of inflexibility
**Context**	Ethical, religious, emancipatory and human-friendly beliefs and values and positive impact of role models, Psychosocial awareness of different age groups, desirable individual characteristics such as patience and peace, love of profession, sympathy, identification with patients and acceptance of criticism	Pre-judgment and different negative mentalities toward patients, occupational barriers such as discrimination in workplace, work overload, financial problems and the effect of colleagues' behavior	Discriminatory beliefs about excluded groups of homeless people, addicts, ethnicities, social status, tendency or lack of tendency for some patients, Undesirable individual characteristics such as not controlling negative emotions and non-acceptance of criticism
**Consequence**	Creating a beautiful image of nursing profession, positive consequence of care	Creating an unpleasant image of the nursing profession, disturbed outcome of caring	Creating an ugly image of the nursing profession, negative outcome of caring

**Figure 1:**
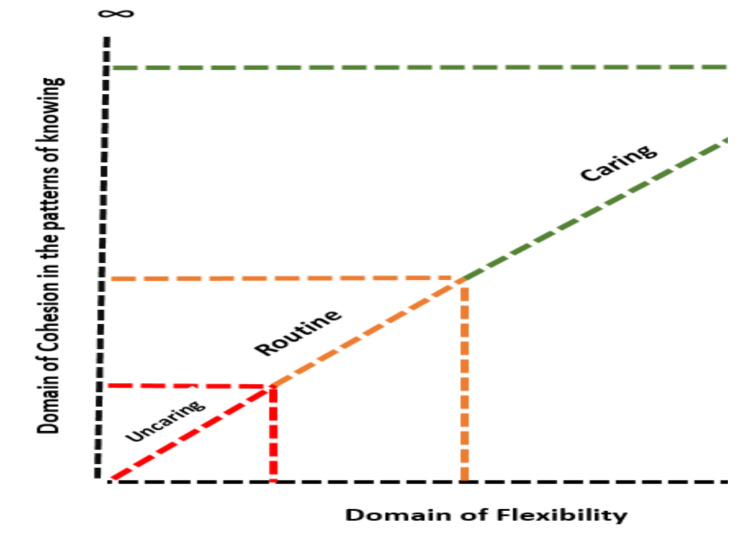
Model of cohesion in the patterns of knowing in domain of flexibility

Flexibility is a variable that belongs to all categories of process (cohesion, domination, and elimination of some patterns of knowing) and connects these three categories to each other like a chain. The diagram of “*The model of cohesion in patterns of knowing in domain of flexibility*” is shown in Figure 1.

## Discussion

The findings of the present study indicated that when nurses apply flexibility along with all patterns of knowing, wholeness and praxis is achieved. From the emancipatory point of view, all patients are equal, regardless of any ethical, racial, ethnic, or cultural attributes, and in this case, cultural security makes sense which means accepting patients on the basis of their own cultural and ethnic framework and avoiding any judgment or prejudice about them (21). Nurses should respect patients' religious, personal, and cultural beliefs, and should try to speak to and listen to patients without any judgment, and make every effort to provide personalized and impartial care (22). The findings indicated that flexibility is related to considering the context of care and applying cohesion in the patterns of knowing. Caring environment and considering the patient's beliefs and values, their involvement, empathy with them and their contribution in decision-making lead to patients' satisfaction, well-being, and creating a therapeutic environment (23). According to the findings, applying cohesion in the patterns of knowing and flexibility is accompanied with therapeutic communication. In this way, nurses use various techniques such as using a sense of humor for effective and therapeutic communication with the patient. A genuine care requires good therapeutic communication (24).

The findings of this study showed that, in critical situations with a lack of time and shortage of resources to save the life of patients, nurses, especially experienced nurses perform some interventions such as prescribing medication that is legally prohibited in normal circumstances. According to the result of the study, clinical experience and records of years are directly and significantly related to the practical competency and use of intuition in clinical decision-making (25). Experienced nurses are more willing to take risk and cross the authority of physicians and are more likely to make non-nursing decisions and even oppose physicians' decisions (26). The findings of the present study indicated that although appointment hours are defined beforehand and are restricted or even prohibited in some wards, such as Intensive Care Units, nurses in various cases allow the patient's family or his/her friends to meet him/her, depending on patient's conditions. The results of a mixed method study indicated that in case of flexibility in meeting time and increasing the hours of meeting and removing the restrictions, satisfaction of the patient and his/her family will increase in ICU (27).

The findings of the present study indicated that inflexibility of the nurse lead to domination of personal knowing and provision of self-centered care. In this case, the nurse is overly insistent on personal values, timing and domain of duties and routines. There may be a semantic link between the concept of self-centeredness and arrogance. Because arrogance means self-superiority in the sense that one considers own values to be superior and more valuable than others are and thinks s/he does not need others to respect him/her, and regards him- herself as not needing their advice and feedbacks (28).

According to the findings of the present study, inflexibility may leads to discriminate between patients and eliminate social justice from the patterns of knowing. Most discrimination is based on patient's social status, commendation of others, and patient's feedback. The results of a study (29) indicated that patients with lower socioeconomic status, immigrants as well as women were discriminated. Discrimination in these groups also depends on age, gender, and education of help-seekers. Recall bias from study participants during the data collection and lack of generalizability of the findings to other geographic, due to small sample size and participants characteristics are the limitations of our study.

In conclusion, according to the findings, applying patterns of knowing along with flexibility, have spectrum so that the least flexibility results in the least cohesion in the use of patterns and consequently inappropriate care behavior, and in the opposite, the most flexibility creates the most cohesion in the use of patterns and care behavior. These spectra are infinite.

## References

[1] Carper BA (1999). Fundamental patterns of knowing in nursing. Perspectives on philosophy of science in nursing: an historical and contemporary anthology.

[2] Archibald MM (2012). The holism of aesthetic knowing in nursing. Nurs Philos.

[3] Billay D, Myrick F, Luhanga F, Yonge O (2007). A pragmatic view of intuitive knowledge in nursing practice. Nurs Forum.

[4] Holtslander LF (2008). Ways of knowing hope: Carper's fundamental patterns as a guide for hope research with bereaved palliative caregivers. Nurs Outlook.

[5] Chinn PL, Kramer MK (2013). Integrated theory & knowledge development in nursing-EBook.

[6] Carnago L, Mast M (2015). Using Ways of Knowing to Guide Emergency Nursing Practice. J Emerg Nurs.

[7] Benner R Patricia, Tanner R Christine, Chesla R Catherine (2009). Expertise in nursing practice: Caring, clinical judgment, and ethics.

[8] Guadron MB (2008). Identification of patterns of knowing used by rural community health nurses in decision-making.

[9] Baixinho CL, Ferraz IC, Ferreira ÓM, Rafael HM (2014). The art and learning patterns of knowing in nursing. Rev Esc Enferm USP.

[10] Sheilds LE (2016). Narrative Knowing: A Learning Strategy for Understanding the role of stories in nursing practice. J Nurs Educ.

[11] Corbin J, Strauss A (2014). Basics of qualitative research: Techniques and procedures for developing grounded theory.

[12] Johnson BM, Webber PB (2005). An introduction to theory and reasoning in nursing.

[13] Speziale HS, Streubert HJ, Carpenter DR (2011). Qualitative research in nursing: Advancing the humanistic imperative.

[14] Polit DF, Beck CT (2009). Essentials of nursing research: Appraising evidence for nursing practice.

[15] Grove SK, Burns N, Gray J (2012). The practice of nursing research: Appraisal, synthesis, and generation of evidence.

[16] Denzin NK, Lincoln YS (2011). The Sage handbook of qualitative research.

[17] Holloway I, Galvin K (2016). Qualitative research in nursing and healthcare.

[18] Scott KW (2004). Relating categories in grounded theory analysis: Using a conditional relationship guide and reflective coding matrix. Qual Rep.

[19] Scott KW, Howell D (2008). Clarifying analysis and interpretation in grounded theory: Using a conditional relationship guide and reflective coding matrix. Int J Qual Methods.

[20] Lincoln YS, Guba EG (1986). But is it rigorous? Trustworthiness and authenticity in naturalistic evaluation. New Dir Prog Eval.

[21] Richardson A, Yarwood J, Richardson S (2017). Expressions of cultural safety in public health nursing practice. Nurs Inq.

[22] Mendes A (2018). Personal beliefs, culture and religion in community nursing care. Br J Community Nurs.

[23] McCormack B, Dewing J, Breslin L, Coyne-Nevin A, Kennedy K, Manning M (2010). Developing person-centred practice: nursing outcomes arising from changes to the care environment in residential settings for older people. Int J Older People Nurs.

[24] Wiechula R, Conroy T, Kitson AL, Marshall RJ, Whitaker N, Rasmussen P (2016). Umbrella review of the evidence: what factors influence the caring relationship between a nurse and patient?. J Adv Nurs.

[25] Miller EM, Hill PD (2018). Intuition in Clinical Decision Making: Differences Among Practicing Nurses. J Holist Nurs.

[26] Maharmeh M, Alasad J, Salami I, Saleh Z, Darawad M (2016). Clinical decision-making among critical care nurses: A qualitative study. Health.

[27] Mitchell ML, Aitken LM (2017). Flexible visiting positively impacted on patients, families and staff in an Australian Intensive Care Unit: A before-after mixed method study. Aust Crit Care.

[28] Hareli S, Weiner B (2000). Accounts for success as determinants of perceived arrogance and modesty. Motiv Emot.

[29] Kruse A, Schmitt E (2016). SoSocial inequality, health and nursing care in old age. Bundesgesundheitsblatt, Gesundheitsforsch ung Gesundheitsschutz.

